# Quantitative carotid MR/PET imaging: comprehensive comparison of MRAC and CTAC attenuation maps in MR/PET emission data and PET/CT

**DOI:** 10.1186/2197-7364-1-S1-A70

**Published:** 2014-07-29

**Authors:** Jason Bini, Philip M Robson, Claudia Calgano, Mootaz Eldib, Zahi A Fayad

**Affiliations:** Translational and Molecular Imaging Institute, Icahn School of Medicine at Mount Sinai, NY, NY USA; Department of Biomedical Engineering, The City College of New York, NY, NY USA

Current MR/PET systems employ segmentation of MR images and subsequent assignment of empirical attenuation map values for quantitative PET reconstruction. In this current study we examine the quantitative differences between our carotid artery imaging protocol on both a PET/CT and an MR/PET scanner controlling for acquisition parameters, such as circulation time, and by replacing the system standard MRAC map in the MR/PET emission with the CTAC map from the PET/CT scan^1^, to directly assess the impact of MRAC methods in the carotid arteries.

All scans were performed with 18F-FDG injections. Eleven subjects were scanned on the Philips sequential MR/PET scanner. Seven of the initial eleven subjects were scanned one week after initial MR/PET acquisition using a PET/CT scanner. Four of the eleven subjects that were initially imaged with MR/PET returned one week after their first scan and were imaged again on the MR/PET. Five comparisons were performed to qualitatively and quantitatively compare PET images from MR/PET and PET/CT acquisitions. The five comparisons performed were: MR/PET MRAC map versus MR/PET CTAC, MR/PET CTAC versus PET/CT, MR/PET with MRAC map versus PET/CT, MR/PET map with coil present versus MR/PET MRAC map without coil present, and MR/PET MRAC map fixed scan-2 versus MR/PET MRAC map fixed scan-1.

MR/PET emission data reconstructed with both the system standard MRAC map and CTAC map, showed excellent correlation (R^2^=0.7970, mean difference 0.03±0.18 SUV, p=0.3382) in quantitative comparisons. Comparisons of percent differences in SUV mean and SUV max within all ROIs analyzed for this initial comparison ranged from -2.6% to 7.4%. (-0.03 to 0.10 SUV).

The results of this study support the use of MR/PET for quantitative measure of metabolic activity in the carotid arteries.

